# Doktor Kot, Doktor Sla – book doctors, plant doctors and the segmentation of the medical market place in Meghalaya, northeast India

**DOI:** 10.1080/13648470.2017.1368830

**Published:** 2017-10-16

**Authors:** Sandra Albert, John Porter, Judith Green

**Affiliations:** a Indian Institute of Public Health Shillong, Shillong, Meghalaya, India; b Public Health Foundation of India, Gurgaon, India; c London School of Hygiene & Tropical Medicine, London, United Kingdom; d Health & Social Care Research, Faculty of Life Sciences & Medicine, Kings College London, London, United Kingdom

**Keywords:** Indigenous, tribal medicine, medical pluralism, Khasi tribe

## Abstract

Despite decades of research on India's plural health care market, the practices of many local health traditions outside the allopathic and codified traditions are under-studied. Drawing on interview and observational data, this paper explores the space in which indigenous traditional Khasi healers in Meghalaya state, northeast India, practice. Khasi indigenous healers describe themselves as *doktor sla*, plant doctors, to distinguish themselves from *doktor kot*, or book doctors. This distinction operates as a rhetorical resource, utilised to carve a distinct sphere of expertise in relation to the allopathic sector, and to mark claims for the specifically local appropriateness of traditional practices within a shifting market of state-sponsored provision. Khasi healers are a heterogeneous group who treat a wide variety of conditions, including physical ailments which have no obvious correlates in biomedical systems, and musculoskeletal disorders, with which they have recognised expertise. In addition to claiming these discrete strengths, healers also present themselves as accommodating deficiencies in biomedicine, including inherent generic weaknesses of allopathic care as well as specific local gaps in rural health care provision. Thus, the expertise niches of traditional healers have evolved through their interactions with, and the needs of, the community, but also through managing a shifting boundary with biomedical practitioners, who are explicitly sceptical of their efficacy, but tacitly accepting of the ways in which they manage the gaps in biomedical provision. While codified non-biomedical traditions in India have engaged in universalising professionalising projects, in this setting at least, non-codified practitioners have instead utilised discourses of localism.

## Introduction

In India, as in many low- and middle-income countries, a majority of health care, particularly for the poor, is typically provided by what are often termed ‘informal’ health care providers (Bloom 2011). In practice, this includes a rather ill-defined group of practitioners with variable training, who are typically not registered with a regulatory body, and largely provide fee-for-service care (Cross and MacGregor 2010; Sudhinaraset et al. 2013). What unites them is that they work on what Pinto (2004) calls the ‘margins of legitimacy’: a shifting boundary, as the formal sector incorporates, subjugates or excludes particular spheres of expertise and practices (Cross and MacGregor 2010). From a policy perspective, this sector is largely framed as a problem, in that it either demonstrates deficiencies in the provision of state-sanctioned services, or provides sub-standard care (Rao et al. 2011; Sudhinaraset et al. 2013; Bloom et al. 2011). However, as Cross and MacGregor (2010) argue, the practices of boundary making between different market sectors may be as much about shoring up the legitimacy of biomedicine as managing threats to health. This paper considers the boundary between one group of ‘informal’ providers, the traditional tribal healers of the Khasi hills, in Meghalaya, northeast India, and the biomedical sector, to shed light on how expertise niches are constructed within changing health markets and policy environments.

India has long been the paradigmatic example of a pluralistic health care market, with the co-existence of multiple systems of both codified and non-codified ‘traditional’ medicine along with both formally trained and informal allopathic, or biomedical, practitioners (Shankar 1992; Lambert 2012). How these diverse healing practices are best classified is highly contested (Lambert 2012; Attewell 2016; Attewell et al. 2012; Hardiman 2009). The specific mix and availability of practitioners varies considerably across the different regions of the county (Lambert 2012; Prasad 2007; Payyappallimana and Hariramamurthi 2012), and the relative legitimacy of different practices has varied historically in response to the professionalising strategies, shifting state support and alignments of particular political and medical projects (Broom, Doron, and Tovey 2009; Khan 2006; Hardiman 2009). Biomedical practices do not necessarily enjoy hegemonic dominance: Kudlu (2016), for instance, has recently documented the processes of commodification and ‘pharmaceuticalisation’ of Keralan Ayurveda as in part a crafting of legitimate alternatives to biomedicine. Indeed, the codified traditions outside the allopathic sector, now known as AYUSH (Ayurveda, Yoga, Unnai, Siddha and Homeopathy), are being increasingly mainstreamed through national health policy (Albert and Porter 2015). However, political processes of inclusion inevitably have their exclusions (Attewell 2016), and AYUSH practices neither represent all non-allopathic traditions in India, nor are common in all regions of the country (Albert et al. 2015; Prasad 2007). Other traditions, as yet without professional regulation or codified systems of knowledge, are now being referred to as Local Health Traditions (LHT) in Indian policy documents (GoI 2002). They are significant providers of health care in many areas, providing a diverse range of therapeutic interventions, and with changing relationships with both biomedicine and the AYUSH sector (Lambert 2012; Attewell 2016). In Meghalaya state, in northeast India, the indigenous tribal traditional healers of the Khasi and Garo communities are perhaps the largest group of health care providers outside the public sector, particularly in rural areas. However, their practices are largely undocumented, apart from ethno-botanical studies of their medical plant therapies (Rao 1981; Tynsong and Tiwari 2010; Roy et al. 2010), and little is known about how they manage the border with biomedicine.

## The health care market in Meghalaya, northeast India

Of India's population of 1.21 billion, 8.14% are classified as ‘tribals’ (Registrar General & Census Commissioner 2013). Indigenous ethnic groups are notified as Scheduled Tribes as per provisions contained in Clause 1 of Articles 342 of the Constitution of India (NCST 2005). Scheduled Tribes are found across the country, but are mainly concentrated in the north-central and north-eastern parts of the country (Ministry of Tribal Affairs 2013). In Meghalaya, a state in northeast India with a population of around 3 million, 86% of the population are classified as Scheduled Tribes, mainly belonging to the matrilineal Khasi-Jaintia and Garo tribes. The Khasi Hills Autonomous District Council (KHADC), a traditional governance body with constitutional powers to legislate on cultural matters, passed the ‘Protection and Promotion of Khasi Traditional Medicine Act’ in 2011 (KHADC 2011). The preamble to this Act states that Khasi Traditional Medicine is accessible, affordable and efficacious, that it provides opportunities for livelihoods and trade and helps in the conservation of biodiversity. The preamble also avers that Khasi traditional medicine is under threat from depletion of medicinal plants, inadequate documentation and transmission, and domination by other systems of health care.

In rural Meghalaya, the health market mainly comprises a state-funded public sector, providing allopathic medicine, which is free at the point of delivery, and a fee (or goods)-for-service sector of tribal indigenous traditional healers. The private biomedical sector that is increasingly playing a major role in health services elsewhere in the country is small, and relatively confined to the capital city of Shillong (GoM 2009). Prior to the National Rural Health Mission (NRHM) initiative of the government, the public health sector primarily focused on providing allopathic services; however, more recently, there has been a national-level policy of also promoting AYUSH in the region (GoI 2008). However, as yet, AYUSH services have little traction with either local policy-makers or the public in the region (Albert and Porter 2015). A survey of 588 households conducted in 2010 in rural Meghalaya (Albert et al. 2015) identified widespread awareness and use of public allopathic and tribal sectors, with the majority (85%) of households ‘sometimes’ or ‘often’ using tribal healers and 46% having done so in the last three months, and 91% having visited local biomedical services. Few, however, had used any AYUSH services, and most respondents were unaware of Ayurveda or other AYUSH traditions.

In the Khasi hills of Meghalaya, the indigenous tribal traditional medicine practiced is referred to as *nongai dawai* and the medicines and medicinal plants that the healers use are referred to as *dawai khasi*. Knowledge of these plants and other therapies is not codified, but instead draws on ancestral knowledge and inheritance, apprenticeship, a notion called *sap* (meaning potential or talent), and experiential learning (Albert, Porter, and Green 2015). The right of indigenous peoples to their traditional systems of medicine has been affirmed by the United Nations General Assembly, which adopted the United Nations Declaration on the Rights of Indigenous Peoples in September 2007 (United Nations 2008). Article 24 of this resolution states that *Indigenous peoples have the right to their traditional medicines and to maintain their health practices, including the conservation of their vital medicinal plants, animals and minerals*. Given the importance of tribal healing traditions in the health care markets of Meghalaya, and in the context of current policy initiatives to promote both allopathic and, more recently, AYUSH health care in the region, there is an urgent need to understand more about how health care is currently provided by existing healers. Focusing on Khasi healers, this study aimed to explore how traditional healers, practicing *nongai dawai*, carve a space for practice in relation to their border with the state-supported biomedical sector in the region.

## Methods

This study drew on multiple sources of data, including in-depth interviews with Khasi traditional healers (*N* = 24) and policy stakeholders (*N* = 46) (local policy-makers, health administrators and biomedical practitioners); three focus group discussions with traditional healers (including a total of 25 practitioners, of whom 12 were also interviewed individually) and ethnographic observations of healers’ practices, forest herb gardens and clinics. In-depth interviews with policy stakeholders were mostly conducted in English by the first author, and those with tribal healers were largely conducted in their own local language, by trained bilingual local research assistants. The majority of interviews with healers lasted over one hour, and covered career histories, experiences of treating patients, and relations with the wider health market. The interviewees were sampled purposively to include a range of experience and gender from four different districts. Recruitment was informed by extensive discussions before fieldwork with office bearers of grass roots organisation of healers, civil society activists, and local researchers to identify an initial sample of healers who had a substantial practice, and were not operating solely as traditional birth attendants or as occasional practitioners. Snowballing, by asking participants to suggest other healers, was used to identify further individuals.

All interviews and focus group discussions were audio-recorded, transcribed verbatim and then translated if necessary into English by the interviewer. Words with no agreed English translation were retained in original Khasi, with explication in parentheses, and later rechecked in discussion with others in the community. Approximately, half the Khasi transcripts and translations were compared and checked by a bilingual elder to refine the translation. Analysis used a thematic content analysis approach, but with the incorporation of some elements of grounded theory, such as detailed open line by line coding of the first five transcripts, to ensure that the team had explored all potential avenues of analysis (Green and Thorogood 2009). The challenges of analysis using translated data are well documented (Pitchforth and van Teijlingen 2005, Larkin, de Casterlé, and Schotsmans 2007). Here, the close involvement of bilingual indigenous research assistants in the team, together with detailed discussions of particular phrases and words within the focus groups, and with research assistants, elders and other members of the community, was essential for developing the analysis. We have retained Khasi words italicised in transcripts for terms where the English equivalents were most problematic. Transcripts are tagged with a note of the source: in-depth interview with Khasi healer (KH), interview with policy stakeholder (SH) or focus group (FG); gender of speaker (M or F) and participant's number.

## Results

The healers interviewed included both men (17) and women (7), as both genders become healers, with little apparent gender division of health care work: men (for instance) can cater to pregnant women, and perform the duties of traditional birth attendants. Formal education ranged from six who had none at all, to four with a tertiary qualification such as a diploma or bachelor's degree. Most worked from a dedicated clinic. Medicinal herbs were largely gathered from nearby forests, with some travelling further afield to gather herbs, and most (14) in addition sourcing plants from assistants or other suppliers. Their therapeutic practices included primarily plant-based remedies, utilising hundreds of largely locally sourced plants (which were called ‘herbs’), manipulations such as massages with medicated oils or balms and application of herbal poultices. Less often mineral and or animal produce were also used in their preparations by some healers. Tribal healers described themselves as *nongai dawai*, and distinguished their sphere of practice first from that of the local ritual healers, termed the *nongkñia*. A majority of the tribal healers in this study identified their religion as Christian (21/24), an identification that was important in the distinction they made between themselves and the *nongkñia*, who followed an indigenous religious faith:

A *nongai dawai* traditional healer can be from any religion. […] He dispenses medicines to people who need it. He can be a priest or a pastor or an ordinary man it does not matter. But a *nongkñia* does not give medicine but he performs prayers, rituals to find out the cause of the sickness and the cure to it. For example if a patient comes to me and asked me to pray for him, I will pray, but I will not know how to perform rites and rituals. (KH035, M)

### Doktor kot and doktor sla: practicing covertly on the margins of biomedicine

A second, and more pervasive, distinction drawn by Khasi healers was between themselves and those who were known as *doktor kot* or *‘*book doctors’: practitioners recognised as trained in biomedical systems. In contrast to *doktor kot*, with formal medical education and credentials, healers described themselves as *doktor sla*, ‘plant doctors’ who rely on knowledge acquired from plants and the forests. Across the interviews, healers made references to the greater formal knowledge and prestige of those who worked in ‘proper settings’:

Biomedical doctors are *doktor kot* [book doctors] where they learn in a proper setting whereas we are *doktor sla* [leaves/plants doctor] whose knowledge has been passed down from one generation to the other. (FG2 KH028, M)

Yes I have mentioned to you earlier that we consider them as *doktor kot* [book doctors] who knows a lot more than we do. (KH013, M)

A shared understanding that tribal traditions had lower status, and less legitimacy, was evident in the way in which traditional therapies were reported as often hidden from ‘book doctors’ when patients used biomedicine and tribal medicine consecutively or simultaneously. Healers’ narratives suggested a tacit understanding between them and their patients that the benefits of their therapies would not be recognised by the *doctor kot*: thus biomedical doctors tended to be kept in the dark on this multiple help-seeking:

The [biomedical] doctors do not know, when patients go to them and the patients say they are taking the herbal practitioners medicines they become furious. When these patients come back from the doctors, we have to massage them. (KH011, M)

The assumption that tribal medicine would be mistrusted by the formal sector meant that therapies were at times provided covertly. This could be particularly challenging for patients being treated as hospital in-patients:

I go to X hospital [large tertiary care referral centre in the capital city, Shillong] frequently to visit the patients with fractures, I apply my medicine on them pretending I am their relative because I am afraid and timid of the nurses and the doctors finding out I am a herbal [tribal medicine] practitioner. (KH018, F)

This hybridity suggests both porousness of the boundary between their practices and those of the allopathic sector, and the unequal flows across it. This was a border which healers and patients clearly manage to navigate, despite the threats of criticism from biomedical practitioners. In interviews, healers were often careful to acknowledge their respect for the biomedical sector, suggesting cases that did not respond to their therapy were referred on to biomedicine, and reporting on occasion seeking biomedical interventions for themselves or their family. However, they also presented some conditions as ‘belonging’ more or less appropriately to their own or to allopathic spheres. Certain illnesses were discussed as ones for which tribal medicine is the only reasonable, or the preferred, option, and others as ones that were best seen by biomedical doctors. Healers reported undertaking an initial triage at consultations to ensure that patients are consulting appropriately:

It depends on what kind of a condition one is suffering from. Some conditions can be cured by using Khasi herbal medicine (*dawai khasi*) whereas some conditions are cured by using foreign/ biomedicine (*dawai phareng*). If I understand that I cannot treat them, I send them to others accordingly. (FG2, KH030, F)

### Market segments and expertise niches

To understand these claims of appropriate allocation of conditions to different health providers, we mapped out the various contexts and situations in which healers reported either treating or referring patients, and the types of problems that they, and others, considered were within their expertise niches. In the context of a more legitimate biomedical sector, tribal healers first claimed to occupy a back-up ‘second stage’ of help-seeking, for problems that were either not solved by biomedicine, or for which a second opinion was sought for confirmation. Second, healers (and others) reported that they were considered a first port of call for a number of conditions, including some for which their expertise was widely known to be superior, and others which were not treated by biomedicine, either because of its inherent exclusions, or because there were more pragmatic local constraints in access.

Khasi healers reported treating a wide variety of disorders. From lists elicited at interview and described in their narratives about patients, these disorders included stomach problems, diarrhoea, skin diseases, boils, abscesses, bites and stings, fevers including malaria, injuries, jaundice, cancers, childhood ailments (e.g. *niañgsohpet*), pregnancy-related ailments, ‘intestinal fevers’, piles, intestinal prolapse, strokes, high blood pressure, kidney stones, urinary tract infections, infertility, fractures, slipped disc, and other musculo-skeletal problems. Healers also described and demonstrated a wide repertoire of treatments, and most operated as generalist practitioners. However, many were recognised (by patients and other healers) for being especially skilled in dealing with certain conditions such as burns, spinal problems or fractures. Even healers who consider themselves ‘well known’ (*tip bha*) as specialists in particular conditions would also treat other ailments, although many were careful to explain that an initial assessment of the patient (using observation, questioning and physical examinations) would be used to decide whether they should more appropriately be sent to the formal sector:

But one thing is that I don't lose hope because from the first visit just by feeling and within two minutes I can understand whether I can help that patient or not and I don't lose hope easily and I even tell my patients that they will be cured. But if I understand that I cannot help them, I send them to allopathic doctors. (KH005, M)

### Problems for which traditional healers are the first port of call

Some conditions were prime candidates for *doktor sla* because they had no obvious equivalent in biomedicine. These conditions were commonly referred to by their indigenous terms. For example, *lait thied sohpet* was a term which healers expected the indigenous research assistants to be familiar with and understand. And they did – but they struggled to explain (to the lead researcher) what exactly it meant, stating that this was a condition well known in Khasi culture. Loosely, the term described a condition of the navel affecting the nerves, which occurs specifically when a person lifts something heavier than their own body weight. Other examples of conditions which were challenging to translate were *ka bih*, often referring to a poison or toxin, and *jingshit ha kasnier* which can be translated literally as ‘fever of the intestines’. The diagnosis of *jingshit ha kasnier* could be made by exclusion, after patients consult biomedical practitioners with symptoms, but fail to recover. As this healer explains, the success of herbal treatment demonstrates the diagnosis:

*jingshit ha kasnier* [fever of the intestines], I send them to the [biomedical] doctors and the doctors try to figure out with their tools what a person is suffering from, but they cannot figure out. But for us traditional healers we have the talents and we try to figure out what the person is suffering from. If we suspect a person having *jingshit ha kasnier*, we treat the patient with the herbs for that particular disease. If there is any kind of relief and improvement in the patient, then that means the person had *jingshit ha kasnier* […] Yes, in order to search for the truth we take the help of herbs. (KH004, M).

In a similar vein, a commonly cited condition for which traditional treatments were sought was *niañgsohpet*, a problem of infancy or childhood, which was described as covering symptoms that might indicate conditions such as infantile colic, indigestion or diarrhoeal disease in early childhood. Again, this was considered curable only with traditional therapies:

Us rural people do not use foreign medicines [biomedicine] especially for *niañgsohpet* we give our own herbal medicines. (KH011, M)

In the focus group discussions, healers’ understandings of *niañgsohpet* were explored in more detail. It emerged as multiple and complex, with difficulties in delineating agreement over symptoms, categories and precise treatment regimes. As the following extract shows, despite debate around the typologies, agreement centred on the appropriateness of traditional treatments, orientated towards the underlying cause, rather than biomedical therapies which might treat symptoms, but not the condition itself:

**KH029 F:** When a baby passes a stool, some of it is green in colour, in some it is watery in nature, in some blood is mixed along with it… If you go to [an allopathic] doctor, they will tell you that it is dysentery. If it is really dysentery, how can the baby survive? But if you go to a traditional healer they will tell you that it is the *niañgsohpet* and they have their own names, *ba saw* [red], *ba jyrngam* [green] and they also have different medicines for each of them.

**KH028 F:** *Niang saw* [red], *niang stem* [yellow], *niang iong* [black].

**KH030 M:** The *niang stem* [yellow] *niañgsohpet* if it is not treated/cured properly, later on it can turn into *jingpang stem* [jaundice], and if the *niang saw* [red] *niañgsohpet* if it is not cured properly, the person can have skin diseases later.….

**KH029 F:** That is why it is important that parents should seek traditional help. If they seek help from [allopathic] doctors, babies will gain weight and look like they are healthy but it can affect them later on in their life. (FG2)

Another group of healers attempt to map the condition onto biomedical notions of aetiology, with the help of a trained birth attendant (TBA). Despite their attempts to explain with reference to (biomedical) doctors’ practices and links to biomedical diagnoses such as TB, they reiterate that biomedical tests are unlikely to find the causative agents, and that biomedical doctors are likely therefore to treat it inappropriately:

**Interviewer:** Ok, let's talk about something else. What is *niañgsohpet*?

**KH016 M:** According to my findings, it is the digestive system that is not functioning properly […]

**KH017 F:** It is the *jakhlia* [unwanted/impure substance] that enters the baby's mouth during birth. There is something in the baby's mouth and it enters into his system while opening his mouth for the first time while crying. We do not have a name, therefore we called it *niañgsohpet*. But traditional birth attendants know about it very well.

**KH019 F:** I am a TBA and to explain it to you, especially in English, is very difficult. At the time of birth, when you look into the baby's mouth you will find something is there. Doctors advise us to take a clean muslin cloth and wipe it. If you apply any kind of medicine or if you do not know how to wipe it, then the baby will swallow it, which can later form other kinds of diseases like TB glands and many others if not treated properly.

Multiple respondents: Yes, yes. [….]

**KH020 F:** The main thing is from the liver that causes the stool of the baby to become green or yellow. I believe in the Khasis when they say *khniang* [insects/organism causing diseases] but if a [biomedical] test is done, they cannot find any *khniang*, and if they find anything wrong they start giving the baby antibiotics. Six months they have to take, how can a small baby take in so much of medicine? (FG3)

Clearly, *niañgsohpet* was difficult to express in terms that would translate to biomedicine, and healers struggled in these attempts to link their explanatory frameworks with those they understood from encounters with the biomedical sector. In short, *niañgsohpet* was one of a number of conditions considered unlikely to be adequately treated by allopathic practitioners simply because it did not fit it into biomedical nosology. For such conditions, healers could claim a near-monopoly of legitimate treatment regimes.

**Figure 1.**
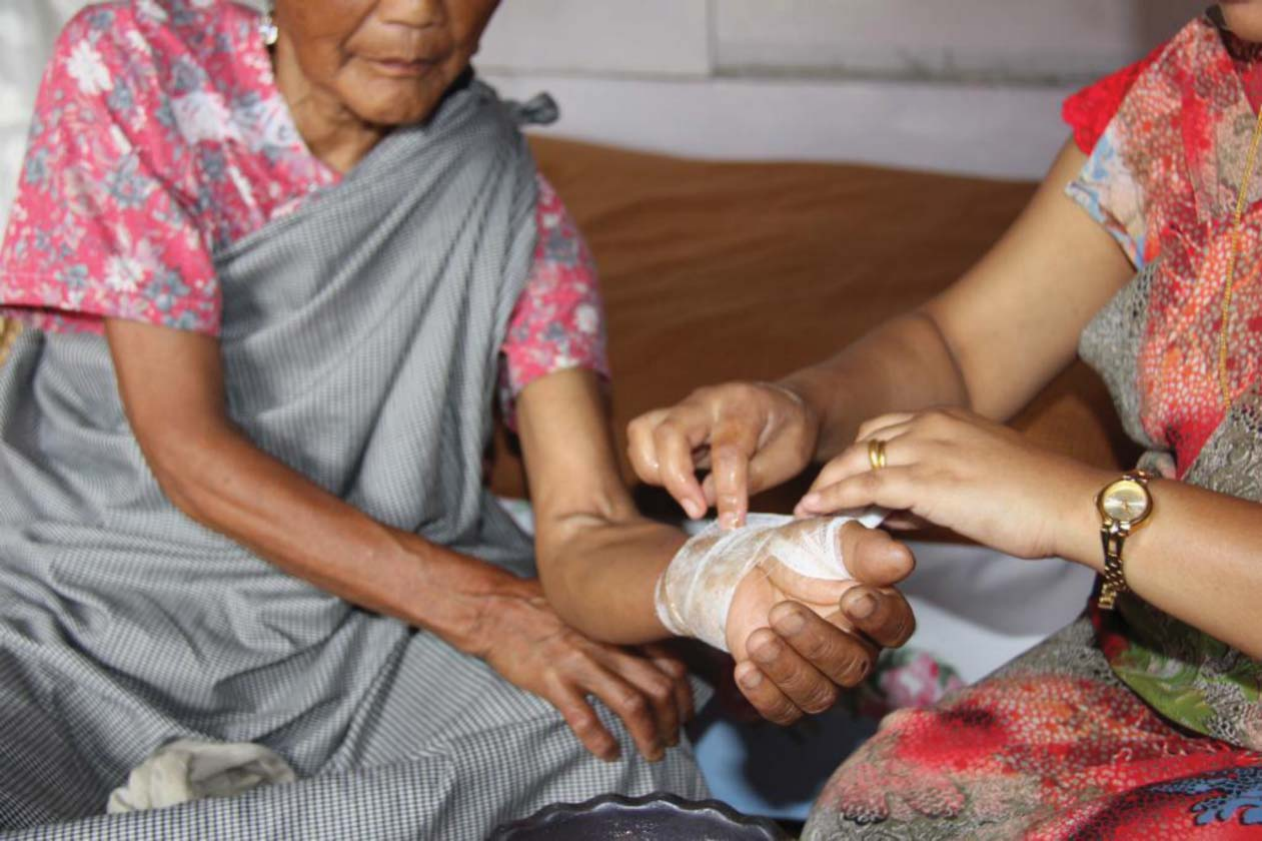
Healer tending to a fracture in an elderly woman.

A second group of conditions for which traditional healers might be preferred was those for which they have a recognised superior expertise. The prime example here is the management of fractures and other musculoskeletal disorders. Fractures were diagnosed with massage techniques, then set using an emulsion of a teaspoonful of a powdered tree bark, gently applied to the patient's wrist. A roll of gauze binds the wrist as the emulsion is applied to each layer of the gauze (Figure 1). Eventually, on drying, the material hardens to something akin to a light plaster cast. It was not only the healers that made claims for their own expertise for musculoskeletal problems: interviews with policy stakeholders also cited the superior skills of Khasi healers, and indeed some reported seeking treatments themselves, either in childhood, or more recently, as this senior bureaucrat describes:

The advice I was getting was that, why don't you look at traditional healer, they said it would set it [damaged foot] straight, by using a sort of a massage therapy and putting some local ointments and all that. So since I envisaged that there was no side effect, there was no harmful effects, so why not try it … so I used it and I felt good at the end of the day, at the end of the treatment, so I'm quite okay now. (SH028, M)

In interviews, both biomedical doctors and local health department policy-makers were at times dismissive of the efficacy of traditional medicines: as one doctor said, ‘we have to go by documented proof (of efficacy). I have not, have not seen so far’ (SH030, M).

However, when it came to fractures, many not only acknowledged patient preferences for tribal healers, but also their efficacy:

They [traditional healers] will do their massage, align the bone or if it is dislocation put it back and then they'll give some, apply some medicine and it really heals very, very fast. (SH032, F)

Community preferences were also cited by healers, if not local policy-makers, for another category of conditions for which they might be a first port of call: those that potentially required surgery, which was widely reported as particularly feared locally. Healers acknowledged in interviews that surgical intervention might not be avoidable, but they also described a range of measures used to manage certain conditions without the necessity for biomedical surgical interventions. Abscesses, for instance, could be managed without incision by herbs:

Here in my village most of the people prefer traditional medicine especially for *thung* [kind of an abscess] or for *bampong* [cancer]. If there is an abscess and pus started forming around it, I treat by applying herbs and gave the patients some medicine to take it orally and all the pus will come out by itself and the patient gets cured. (KH036, F).

### Filling in the gaps of biomedicine

If traditional healers had particular recognised niches of expertise, for conditions that were agreed as more appropriately or preferentially allocated to the traditional sector, they also treated a large number of ailments for which patients reportedly considered, or preferred, allopathic treatments if they were available. For these, Khasi healers presented themselves as primarily filling ‘gaps’ in state-provided biomedical provisions. This included gaps that emerge from problems of access, where (in rural areas at least), biomedical services might be unavailable, particularly in emergencies at night, or in remote areas:

But for us especially in the rural areas we are there just to aid them, so that they can reach the hospital and not die half way. (KH008, M)

Most of the people who think that the case is very serious they will take traditional medicine only for some relief or improvement until they reach the hospital. For example if it was at night and somebody is sick they will come to my house asking for medicine but early in the morning they will go to the hospital for treatment. (KH036, F)

One particularly complex case, where allopathic services would have been the first choice of the healer, was reported of a patient who needed expertise unavailable locally:

I examined the child and told the parents that I could not take any chances, because this was my first experience with such a case and if anything goes wrong the child might die….The child did not have skin over his abdomen and his entire intestines could be seen. [….] The mother took the child to Guwahati Medical College for treatment. (KH008, M)

The parents were then referred from the local clinic to a specialist tertiary care centre outside the state, but were unable to travel there, so the healer reported reluctantly taking the case on, with eventual success (as he demonstrated by showing photographs).

Other gaps in biomedicine related to what were presented as inherent failures of the system itself, rather than gaps in local health provision. In such cases, traditional healers described themselves as being the last resort for those who had failed to find cures or were ‘fed up’ with ineffective biomedical therapies. The most extreme gap that traditional healers dealt with were those patients who had reached the end of what biomedical services could provide, such as those requiring palliative and terminal care, or those still suffering for whom no allopathic diagnosis could be found. For those who have lost hope in biomedicine, or who have been told that nothing more could be done in biomedical settings, healers reported providing care to ease suffering. Thus, palliative care was also a provision not made (currently) by local public services:

Still there are people who don't come to us [initially]. They come when all doors have shut for them, then they come to us, so it is very late then. There are cases that are very, very late. (KH019, F)

Only few patients come to me directly with their problems without first seeking any other medical help. (KH004, M)

Given the marginality of their practice in relation to biomedicine, though, within such hybrid systems of help-seeking, healers were often careful to position their contribution as essentially complementary, in that it could be used to ameliorate the inevitable iatrogenic disorders of biomedicine. For chronic ailments and convalescence periods, for instance, herbal medicine and biomedicine were reportedly often used together, with herbal medicine used to help the patient recover, while biomedicine tackled the disease. One healer discussed this in the context of cure and recovery from tuberculosis (TB) and its treatment:

I told my patient to visit the CHC [community health centre] and have her sputum tested because it might be a symptom of TB. There are some patients who will not go and insist on me treating them. I tell them that it is better to go to the hospital for a thorough examination and if the reports are positive, then at least for six months you have to continue those medicines provided by the CHC because allopathic medicine are also important and they can cure certain diseases which herbal medicine would take a long time to cure. But there are also some patients who came back to me for treatment after taking allopathic treatment for six months because they say that the medicines prescribed to them are too strong and they feel very weak. (KH013, M)

Again, this account suggests working both against and within the greater legitimacy of biomedical services for conditions such as TB, where accepting a patient for treatment has to be justified (to the interviewer, at least) as resulting from the patient's ‘insistence’. But the underlying rationale here, the healer suggests, is that his therapies ameliorate the enervating effects of biomedical remedies. Beyond such claims for the complementarity of traditional healing were claims for its ‘thoroughness’, in eliminating diseases from their ‘roots’, so as to ensure no recurrence of symptoms. In contrast, biomedical practitioners might only achieve a partial cure, because the roots of the disease persisted, from poison or toxin (*ka bih*) remaining in the body, untouched by biomedical therapies:

**KH020:** One thing it is also that Khasi medicine can cure diseases right from its roots.

**KH007:** Yes from its roots.

**KH020:** Most of the time doctors prescribed painkillers just to provide temporary relief, but Khasi medicine if you are regular in taking it, it can cure right from its roots.;

**KH014:** Yes, yes. (FG3)

### Flows across the boundary: referrals and investigations

Overall, then, within a hybrid system of health care, some conditions were considered to be the primary province of either allopathic or traditional healers, but most could in practice be treated by combinations of therapies and healers in sequence or simultaneously. This raised challenges of dealing with potentially conflicting requirements of the different systems. Healers were aware of some of the formal protocols used in the allopathic sector, but claimed that some were not in line with community preferences. Home births, for instance, were preferred locally, although government protocols encourage hospital deliveries. Healers therefore had to manage patients in the knowledge that their own therapies are not simply held in low regard, but might also be actively discouraged:

But, Dr. X [names gynaecologist from hospital in capital] told us that you should not massage a pregnant woman, but people in our place especially pregnant woman seek it and come for a massage. Other doctors, a paediatrician told us that if a child has diarrhoea/dysentery and vomiting, you should take the child immediately to the hospital, but we give the child herbal medicine and the child is cured. (KH036, F)

Indeed, the codified knowledge bases and practices of *doktor kot* loomed large in the accounts of healers. Known departures from these bodies of orthodoxy were variously defended on the grounds of greater efficacy (in the examples above of specific local conditions) or, as here, by reference to local tradition and community norms. But if *doktor kot* was a constant referent for the healers in accounts of their own practice, there appeared to be little direct interaction between healers and biomedical doctors, and few opportunities for formal referral between the sectors. One exception was of referrals made in cases of suspected TB, which were encouraged through government-sponsored awareness programmes conducted as part of the DOTS (Directly Observed Treatment, Short course for TB) initiative, as well as the work of the local university, which had recently organised training workshops as part of a pilot project to training healers to become DOTS providers in rural areas. Healers reported that they had been eager to learn from this opportunity, but a few narrated conflicts with biomedical doctors at such meetings, and experiences of disrespect stemming from biomedical disdain for their knowledge:

I told them “you come here to make us understand but when we are suggesting something, you throw away our suggestions.” … I went there because I thought that it will be a kind of training for us to improve ourselves, but instead as they are more educated than us, they criticise and look down at us. (FG2 KH028, M)

However, apart from these limited, and perhaps less than effective, attempts at providing interchanges between traditional healers and the formal sector, many informal routes of exchange were reported. When tribal healers decide that a patient's problem does not fall within their expertise, they recommend alternative sources of help by making an informal referral. Healers reported ‘sending’, or referring patients to other healers with specific specialisms, but also to biomedical doctors if the case is deemed appropriate, or if they could not effect a remedy over time:

I feel sad when I cannot treat a patient, but I tell them that if you feel that it is not helping you, come and inform me so that I can send (*phah*) you to another *doktor sla* or to a *doktor kot*. (FG2 KH030, F)

And if in a span of three days if the treatment does not help, we tell them to seek help [*pyrshang*] from others and not to waste their time and money with us. If I understand that I cannot treat this particular condition I tell them to go and visit doctors who are specialized in that particular condition like those *doktor kot*. (KH013, M)

The use of tests and investigations was a second informal space of interchange across the boundary with biomedicine. Directly (verbally) referring patients for investigations, although not frequent, did occur under certain circumstances, but reports of investigation were referred to in many healers’ narratives, with some keeping copies of test reports brought in by patients. Investigations that allow a healer to ‘see’ the pathology within, such as X-rays for fractures and scans for kidney stones, were especially appreciated, but healers pointed out such tests were sometimes requested to reassure patients, rather than to aid diagnosis:

Yes, there are lots of patients that I send for further testing in the hospitals because they do not believe me because I used only my hands and they are scared because they think that my diagnoses might be wrong especially for cyst, tumours and even stones. But if they bring me the report it is exactly what I told them. So first I tell them about what I found out just by touching and looking and if they do not believe me, then I tell them on the same day to have themselves tested in hospitals. (KH010, M)

Like referrals, these requests were informal, and healers felt that biomedical practitioners were unlikely to be aware that many patients were referred for treatment or tests by healers, as they were made orally, unaccompanied by any written notes.

Yes, I do send patients to doctors and even to Shillong [capital city]. But they [doctors] do not know that we sent them because we do not give them [the patients] a slip saying that this patient has been sent by me to you for treatment or go for an X-ray and scanning in this hospital. (KH016, M)

Thus, there were several routes of exchange with biomedical services, arising from both recognition of different niches of expertise and the tacit understanding that biomedical tests might confer legitimacy, but these were, in general, not formally recognised as such by the biomedical sector.

## Discussion

Cross and MacGregor (2010) note that expertise in health, like any expertise, is located within a context of ‘social institutions, moral universes and networks of power’. In Khasi society, this context is a pluralistic health sector in which tribal medicine has marked out a niche of distinct, but overlapping, services from the publically funded and state-legitimated biomedical sector. Traditional providers frequently reference this sector in describing their own sphere of practice, interpolate their therapeutic regimes with the material goods of biomedicine (for instance, through using tests and making referrals) and they explicitly demarcate their role as providing for both gaps and deficiencies in biomedicine. However, they also make claims to provide for a series of conditions which lie outside the borders of biomedicine, including some ailments for which even the biomedical elite will seek ‘traditional’ care. Within this plural market of hybrid treatment regimes, the state-provided biomedical system has considerably more legitimacy and prestige. Healers in Meghalaya have to carve out their space for practice with reference to this system of *doktor kot* practitioners, and with a clear awareness of their own marginal position as *doktor sla*. As Hardiman and Mukharji (2012) describe, such ‘subaltern’ traditions stand in complex relationships to dominant traditions, with ‘a plethora of micropolitics that constantly evade the control of the molar (i.e. centralising power)’ (2012, 17). Unconstrained by the dogmas of codified traditions, healers manage the flows and boundaries between biomedicine in contingent and multiple ways: variously resisting, ameliorating, and co-opting. Like other subaltern healers, reported from India (Lambert 2012) and elsewhere (Hinojosa 2002), the relationships they negotiate both (at times) directly with *doktor kot*, but more typically on behalf of their clients, are framed within a fragile dialogue prone to disdain on the one hand, and subjugation on the other. This subaltern status was tied, though, to that of their clients: rural communities who suffered with ailments that were not only untreatable by biomedicine, but not even recognised by biomedicine, and whose access to biomedical services was at times constrained by their rural location.

**Figure 2.**
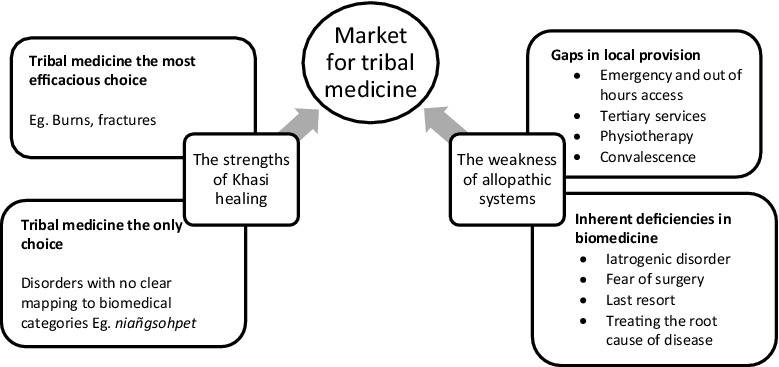
Creating a space for traditional healing on the margins of biomedicine.

In summary, Khasi healers carve out their sphere of practice by both delineating the particular strengths of Khasi herbal traditions, and by claiming to offset the weaknesses of local state-provided services in particular, and of allopathic practice in general (see Figure 2). They describe their expertise, and subsequent market niche, as formed and managed on the borders of biomedicine, as they triage appropriate conditions for treatment, and present themselves as ameliorating gaps in biomedical services, such emergency care, or palliative care. They make stronger, if less explicit, claims for treating a set of conditions which were well known locally, but which could not easily be translated into biomedical categories. Disorders such as *niañgsohpet* and *lait thied sohpet* were illnesses that were broadly understood within the community, with no ready translations for the Khasi terms in English, nor any biomedical equivalent. Claims for managing the general and specific deficiencies of allopathic care resonate with the segmentation practices of Ayurvedic practitioners discussed by Nisula (2006), in Mysore, where Ayurvedic healers carved a space in managing the ‘failures’ of (an often first choice) biomedical system and wanted further integration to survive in a plural market. There are also resonances, at a smaller scale, of historically shifting Ayurvedic claims to antiquity, unique appropriateness for a (national) clientele and (later) globalisation, in the claims of providing a universalist alternative to biomedicine (Hardiman 2009). However, in contrast, the recent globalising claims of Ayurveda, Khasi traditions were self-consciously wrought as specifically ‘local’, with diagnoses and therapies presented as intrinsic to a locally resident clientele, not to any globalising or universalist nosology or market.

Thus, Khasi healers adapt to the (current) availability and legitimacy of biomedical services within the community, working at and across its margins, and filling in the gaps in services offered by the public sector. With and (as yet) un-codified system, they continue to provide a substantial proportion of rural health care. Thus far, this has been provided at the margins of the public health system and outside state recognition. The need to develop improved understanding of the informal sector and the medical market they serve has been highlighted in recent papers (Bloom, Standing, and Lloyd 2008; Bloom et al. 2011). This study has suggested, though, that expertise niches in plural markets like those of Meghalaya are likely to be in constant flux, as they are negotiated across shifting provisions of biomedical, and more recently AYUSH, therapeutic services. For healers such as those from the Khasi community, claims for legitimacy currently rest on specificity and localism, rather than any universalist claims. As the orthodoxies of more dominant sectors change in, for instance, recruiting healers within the DOTS programme, or advocating for AYUSH integration (Albert and Porter 2015), healers have to adapt their own positions. Thus, at times, they stressed their complementary role in health care, in mopping up demands which were not or could not be met; at times, they framed their practice as explicitly resistant, in providing a (locally) appropriate service for problems which were not, and could not be, recognised by *doktor kot*. Resistance was of necessity covert: treating hospital patients under the radar, or providing local remedies (such as massage for pregnant women) which were known to be counter to prevailing biomedical wisdom. Community preferences were offered as rationale, with a claim for localness, and tradition, bolstering the attempt to carve legitimate areas of practice. Formalising traditional practice in Meghalaya might improve the status of local healers in relation to the public sector, and would recognise a sector with more resonance for local communities than AYUSH systems, which are little used in the region. However, any such formalisation is likely to be contingent and fragile, as what constitutes ‘traditional’ practice is clearly shaped by the deficiencies, margins and inherent weakness of the allopathic sector as much as the particular strengths of indigenous therapeutic traditions.
